# Ten-year clinical and radiological outcomes with a vitamin E-infused highly cross-linked polyethylene acetabular cup

**DOI:** 10.1302/2633-1462.510.BJO-2023-0179.R1

**Published:** 2024-10-03

**Authors:** Yama Afghanyar, Bedjan Afghanyar, Lennard Loweg, Philipp Drees, Erol Gercek, Jens Dargel, Philipp Rehbein, Karl P. Kutzner

**Affiliations:** 1 Department of Orthopaedics and Traumatology, University Medical Centre of the Johannes Gutenberg-University of Mainz, Mainz, Germany; 2 Department of Orthopaedics, St. Josefs-Hospital Wiesbaden, Wiesbaden, Germany; 3 ENDOPROTHETICUM, Mainz, Germany

**Keywords:** Hip, Arthroplasty, Vitamys, EBRA, Migration, Wear, highly cross-linked polyethylene, radiological outcomes, acetabular components, osteolysis, revision surgeries, aseptic loosening, acetabular cup, strength, titanium, Harris Hip Score

## Abstract

**Aims:**

Limited implant survival due to aseptic cup loosening is most commonly responsible for revision total hip arthroplasty (THA). Advances in implant designs and materials have been crucial in addressing those challenges. Vitamin E-infused highly cross-linked polyethylene (VEPE) promises strong wear resistance, high oxidative stability, and superior mechanical strength. Although VEPE monoblock cups have shown good mid-term performance and excellent wear patterns, long-term results remain unclear. This study evaluated migration and wear patterns and clinical and radiological outcomes at a minimum of ten years’ follow-up.

**Methods:**

This prospective observational study investigated 101 cases of primary THA over a mean duration of 129 months (120 to 149). At last follow-up, 57 cases with complete clinical and radiological outcomes were evaluated. In all cases, the acetabular component comprised an uncemented titanium particle-coated VEPE monoblock cup. Patients were assessed clinically and radiologically using the Harris Hip Score, visual analogue scale (pain and satisfaction), and an anteroposterior radiograph. Cup migration and polyethylene wear were measured using Einzel-Bild-Röntgen-Analyze software. All complications and associated treatments were documented until final follow-up.

**Results:**

Clinical assessment showed persistent major improvement in all scores. On radiological assessment, only one case showed a lucent line (without symptoms). At last follow-up, wear and migration were below the critical thresholds. No cup-related revisions were needed, indicating an outstanding survival rate of 100%.

**Conclusion:**

Isoelastic VEPE cups offer high success rates and may prevent osteolysis, aseptic loosening, and the need for revision surgeries in the long term. However, longer follow-up is needed to validate our findings and confirm the advantages offered by this cup.

Cite this article: *Bone Jt Open* 2024;5(10):825–831.

## Introduction

Total hip arthroplasty (THA) is a highly successful surgical procedure, and has offered remarkable results over the past century.^1^ Limited implant survival due to aseptic cup loosening is the most common cause for revision.^2-4^ It remains a major long-term concern, and is mainly related to polyethylene (PE) embrittlement and wear-induced bone osteolysis.^5^ Advances in implant designs and materials have been crucial in addressing these challenges.

Developments over the past few decades have particularly focused on PE manufacturing, and have ranged from the creation of ultra-high molecular weight PE (UHMWPE) to the cross-linking of PE for obtaining highly cross-linked PE (HXLPE). The progression from conventional UHMWPE to HXLPE represents a major milestone in joint arthroplasty, as it led to a consistent reduction in wear and revision rates.^6-8^ However, free radical-induced oxidative deterioration resulting from irradiation of PE represents a limitation of HXLPE.^9^ The lipophilic antioxidant, alpha-tocopherol (vitamin E), has been incorporated to minimize oxidative deterioration and provide mechanical fatigue strength.^10,11^ Vitamin E-infused HXLPE (VEPE) promises strong wear resistance, high oxidative stability, and superior mechanical strength.^10,11^ In vitro studies have confirmed significantly superior wear resistance compared to UHMWPE.^8,12^

In addition to improving wear resistance to ensure long-term implant durability, it is essential that stress shielding is reduced to maintain good bone stock for possible future revision. Implants that mimic the elasticity of cancellous bone offer superior load transmission to surrounding bone; this may lower stress shielding in the long term.^13,14^ Notably, isoelastic VEPE cups offer superior preservation of bone mineral density compared with UHMWPE cups.^15-17^

Several studies have demonstrated good mid-term performance with VEPE monoblock cups.^18,19^ Although they offer excellent wear patterns and revision rates,^20-22^ long-term results are unavailable.

The present study primarily aimed to evaluate long-term migration and wear patterns of an isoelastic VEPE monoblock cup. The secondary aim was to evaluate clinical and radiological results, and the tertiary aim was to report implant survival rates at a minimum of ten years’ follow-up.

## Methods

This prospective observational study evaluated follow-up data for a minimum of ten years; five-year results have been previously reported from the same cohort.^18^ The first 101 consecutive primary THA cases (in 96 patients) between March 2010 and September 2011 were investigated. Patients aged between 20 and 85 years with various indications were included.

**Fig. 1:**
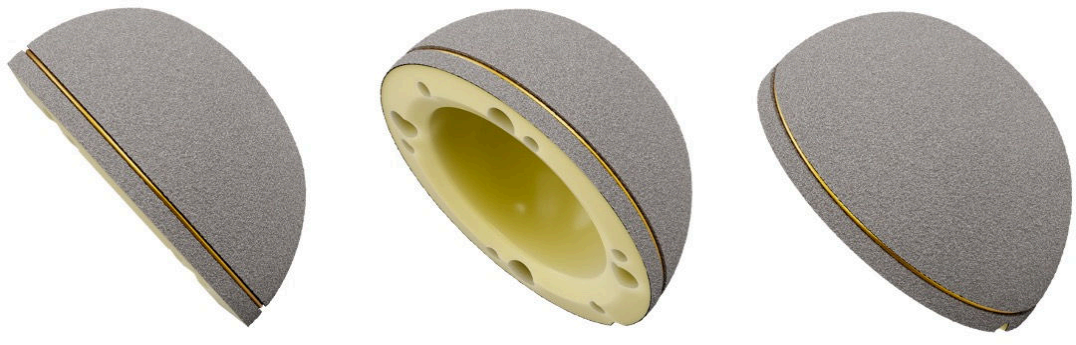
The RM Pressfit vitamys cup (Mathys, Switzerland).

The RM Pressfit vitamys cup (Mathys, Switzerland) was used as the acetabular component in all cases (Figure 1). This preassembled uncemented monoblock cup is composed of titanium particle-coated vitamin E-infused HXLPE. Primary stability is achieved using the equatorial pressfit technique, and secondary stability is ensured by bony integration of the titanium coating. The acetabular component was combined with different uncemented stems, including those from Nanos (Smith & Nephew (UK) (33.7%)); twinSys (Mathys (Switzerland) (27.7%)); optimys (Mathys (17.8%)); Metha (B. Braun/Aesculap (Germany) (12.8%)); Mayo (Zimmer (USA) (3.0%)); Marathon (Smith & Nephew (3.0%)); and ProxyPlus (Smith & Nephew (2.0%)). All patients received a 28 mm ceramic head.

All surgeries were performed by four different consultant surgeons (PR, JD, KPK, LL) via an anterolateral approach with the patient in the supine position. The mean duration of surgery was 56.4 minutes (30.0 to 93.0). Standardized assisted physiotherapy with full weightbearing was permitted on the first day. The patients received standard prophylaxis with etoricoxib (90 mg once daily for two weeks) for preventing heterotopic ossification; dabigatran (150 mg) was administered once daily for four weeks as anticoagulation therapy.

Patients were assessed clinically and radiologically at predefined intervals. Patients were first assessed preoperatively, and then at six weeks, six months, 12 months, two years, five years, and ten years after surgery. Clinical outcomes were assessed using the Harris Hip Score (HHS)^23^ and visual analogue scale (VAS) for pain at rest and on load-bearing (0 = no pain; 10 = worst possible pain).

Radiological assessments were performed using standardized anteroposterior pelvic radiographs. Lucent lines and osteolysis were analyzed based on the criteria described by Engh et al,^24^ and were defined in the zones described by DeLee and Charnley.^25^ A postoperative posterior line behind the cup was defined as a “posterior gap” due to the aspherical cup design. If such a line appeared for the first time during follow-up or increased over time, it was defined as a lucent line.

Acetabular component migration and PE wear were measured using Einzel-Bild-Röntgen-Analyze (EBRA) software (University of Innsbruck, Austria). EBRA is a computer-based software which evaluates standard anterior-posterior pelvis radiographs over time. Predefined reference points were selected on each radiograph (minimum of four radiographs per patient); this allowed for measurement of femoral head and cup migration, and the wear in horizontal and vertical direction. The accuracy of EBRA has been validated within a distance of 1 mm.^26,27^ All measurements were performed by the same observer (BA) to avoid interobserver variations in terms of predefined points and measurements.

All complications and associated treatments were documented until final follow-up examination. Heterotopic ossifications were documented based on the Brooker classification.^28^

All procedures were performed in accordance with the Helsinki Declaration 1964, and all patients provided verbal and written consent for participation prior to inclusion. Ethical approval was obtained (approval number: FF 154/2017) from the Ethics Commission of the State Medical Association Hessen, Frankfurt, Germany. The trial was registered as an observational study at clinicaltrials.gov (NCT04322916).

### Statistical analysis

Statistical analyses were performed using standard descriptive statistics, including the mean, SD, and range. Differences were evaluated using paired *t*-tests; Wilcoxon signed-rank tests were used for non-normal data. A p-value ≤ 0.05 was considered statistically significant. Statistical analyses were conducted using SAS software 9.4 (SAS Institute, USA). The total vector of cup migration was calculated based on the Pythagoras’ theorem using their respective EBRA values in x- and y- axes; total wear (i.e. head penetration) was derived similarly. As the total vector may be masked when considering the average individual migration vectors in either caudal or cranial direction, the distribution of the total vector was analyzed with regard to angular quarters (i.e. 0° to 90°, 90° to 180°, 180° to 270°, and 270° to 360°). Differences in total vectors with respect to quarters were evaluated using the Kruskal-Wallis test. Annual rates were calculated at intervals of three, 12, 24, 60, 84, and 120 months.

## Results

A total of 96 patients (101 hips) were followed-up over a mean duration of 129.3 months (120.0 to 148.9); five patients were treated with one-stage bilateral THA, and 57 cases with complete clinical and radiological follow-up data were evaluated after a minimum of ten years. An additional 14 cases were followed up clinically without radiographs. During the study period, seven patients were lost to follow-up (among whom some refused to attend due to non-hip-related illnesses) and 23 died with the implants in situ. All deaths were unrelated to the surgery and did not occur within the first postoperative year.

The most common indication was primary osteoarthritis (93%); the others included femoral head necrosis (3%), secondary osteoarthritis (2%), fracture (1%), and congenital dysplasia (1%). Details of the baseline demographic characteristics and diagnoses are provided in Table I.

**Table I.** Detailed demographic data.

Variable	Female	Male	Total
Hips, n	63	38	101
Mean age, yrs (range)	70.3 (50.7 to 84.0)	67.9 (51.0 to 84.3)	69.4 (50.7 to 84.3)
Mean BMI, kg/m² (range)	27.3 (19.3 to 39.1)	27.9 (20.2 to 41.5)	27.5 (19.3 to 41.5)
**Diagnosis, n (%)**			
Primary osteoarthritis			94 (93.1)
Secondary osteoarthritis			2 (2.0)
Femoral head necrosis			3 (3.0)
Femoral neck fracture			1 (1.0)
Congenital dysplasia			1 (1.0)

Clinical examination revealed a major improvement in all scores. The mean HHS improved from 49.9 (SD 15.0) at baseline to 96.4 (SD 6.7) at last follow-up (p = 0.052). On the VAS, the mean score for pain at rest decreased from 5.0 (SD 3.3) preoperatively to 0.0 (SD 0.2). The mean pain on load-bearing decreased from 7.7 (SD 2.2) to 0.2 (SD 0.5). Satisfaction increased from 1.7 (SD 2.0) to 9.9 (SD 0.3) after ten years.

**Fig. 2:**
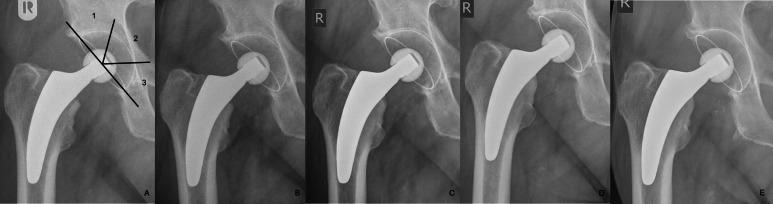
Radiographs of 53-year-old male patient with signs of a lucent line. a) Immediately postoperation with DeLee and Charnley zones, b) six weeks postoperatively, c) follow-up at one year, d) five years, and e) ten years.

Long-term radiographic evaluation revealed a persistent lucent line in one case (53 years old, male patient), which remained unchanged since the mid-term results after five years (DeLee Charnley zone 2 and partially in zone 1) (Figure 2). In this asymptomatic patient, the lucent line appeared at five years and remained stable at ten years. Further radiological assessments showed no evidence of osteolysis, lucent lines, or stress-shielding. Heterotopic bone formation was observed in six cases (10.5%); four cases (7.0%) and two cases (3.5%) showed Brooker I and II ossification, respectively. No further radiological alterations were detected.

**Fig. 3:**
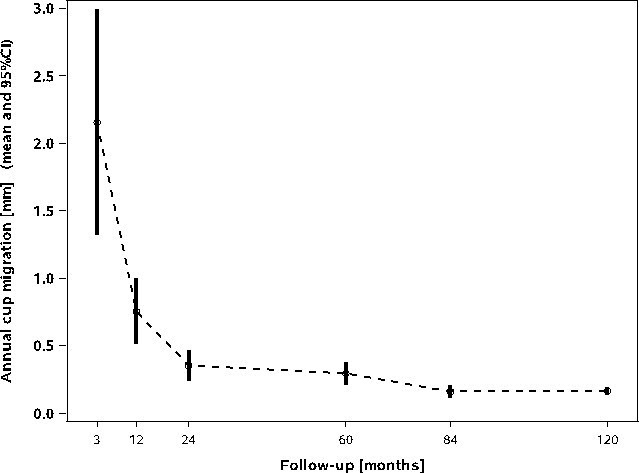
Box plot diagram for annual cup migration rate.

**Fig. 4:**
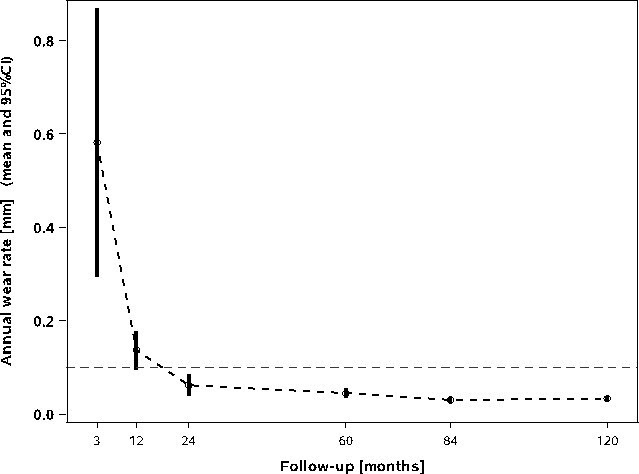
Box plot diagram for annual wear rate.

At last follow-up, 265 of 308 radiographs were available for EBRA measurement. The mean number of radiographs per patient was 5.41 (3.0 to 8.0); the data from 49 patients were used for the final EBRA analysis. At final follow-up, mean cup migration increased to 1.67 mm (SD 1.03; 0.32 to 5.53) from a value of 1.34 mm (SD 0.63; 0.09 to 3.14), which was recorded at five years’ follow-up. During the critical first year, we observed a mean cup migration of 0.80 mm (SD 0.71; 0.07 to 2.81). The critical threshold of 1 mm for cup migration in both directions was exceeded in six patients; however, this settled over the second year in all cases. The annual cup migration rate decreased from 0.30 mm (00.4 to 1.06) at five years to 0.16 mm (0.03 to 0.50) at ten years. However, the cups stabilized after initially showing high migration (Figure 3). The mean total wear at last follow-up was 0.35 mm (SD 0.21; 0.00 to 0.87); the measured values decreased slightly from the five-year results (at 0.40 mm (SD 0.24; 0.03 to 1.00)). Creep and wear during the initial 12 postoperative months were measured to be 0.18 mm (SD 0.17; 0.00 to 0.78). At last follow-up, the mean annual wear rate was 0.03 mm (SD 0.02; 0.00 to 0.08) per year (Figure 4).

A total of ten complications were recorded during the ten-year follow-up period. These included haematoma formation, periprosthetic fractures, femoral nerve palsy, and superficial infection in the early postoperative period in four, two, one, and one cases, respectively. These complications were treated successfully with conservative approaches, and the case of superficial infection was treated using antibiotics. An intraoperative fissure of the femur was observed in two cases; this healed uneventfully after cerclage wiring. The sensory paralysis of the femoral nerve had completely resolved at the first follow-up visit. During further follow-up, two cases demonstrated traumatic periprosthetic fractures of the femoral component without involvement of the acetabular component; both were treated with cerclage wiring and stem revision. At last follow-up, none of the patients required cup-related revision due to aseptic loosening, mechanical failure, or any other reasons.

## Discussion

To the best of our knowledge, this is the first long-term study on the RM Pressfit vitamys cup since its introduction in 2009. At a minimum of ten years’ follow-up, the implant showed excellent clinical results with considerably high functional scores and satisfaction. Slight migration and wear were observed compared to the five-year results, indicating overall stability of the implant. Further radiological alterations were minimal and, most importantly, clinically uneventful; no signs of cup loosening were observed. No cup-related revisions have been required to date, resulting in an outstanding survival rate of 100% at ten years.

The optimal acetabular component design remains an area of debate. As evident from the German arthroplasty register, cementless cups remain the mainstay of THA.^2^ Despite largely successful long-term outcomes, aseptic loosening is a particular impediment to the survival of acetabular components. The purportedly superior survival rates and lower revision rates of newer cups are believed to be mainly associated with reduced migration and wear.

Although extended cup migration within the initial 12 to 24 months has been shown to be predictive of long-term cup loosening,^29-31^ there is no consensus regarding the critical threshold. Krismer et al^30^ identified cup migration by more than 1 mm within the initial two years to be a predictor of late aseptic loosening. Some other authors mentioned a critical threshold of > 2 mm over the initial two years.^29,32^ In this context, loosening has been defined by an increase in total migration by 0.5 mm per year.^33,34^ The present study detected cup migration by 0.80 mm after the initial (critical) 12 months; after the bedding-in process at 24 months, this value was 0.86 mm. Both results were clearly below the aforementioned critical thresholds, indicating the safety of the cup. Earlier results have suggested good long-term performance of the cup; this was confirmed by an overall mean migration of 1.67 mm at a minimum of ten years and 100% implant survival. Notably, the measured annual cup migration was well below the critical threshold. Although migration could be caused by many factors, minor migration over the years is often asymptomatic.^30,34^

Recent studies have shown similar first year migration rates and stabilization after the bedding-in phase.^22,35^ Additionally, the migration rates observed in our cohort were in agreement with those of comparable studies.^18,22^ However, long-term results are not available from the other studies.

In addition to the migration pattern, the wear rate is a key factor for cup durability. Dumbleton et al^36^ had established the widely used thresholds after reviewing the association with wear-related osteolysis. In their study, osteolysis was rarely found and almost absent with annual wear rates of less than 0.1 mm and 0.05 mm, respectively. In recent years, Elke and Rieker^37^ found that a wear rate of less than 0.1 mm/year correlated with an osteolysis-free survival of less than 20 years across all femoral head sizes. The present study observed a mean annual wear rate of 0.03 mm at last follow-up; this was well below the critical thresholds.

In terms of wear, our findings are consistent with recently published mid-term results. In their randomized controlled trial (RCT), Rochcongar et al^20^ found a significant difference in wear patterns between the RM Pressfit vitamys cup and its precursor cup, RM Pressfit (Mathys) with UHMWPE. On five-year follow-up, the wear rate was 66% lower with VEPE cups than with UHMWPE cups. On six-year follow-up of 199 patients, Massier et al^21^ also confirmed significantly superior wear performance with the RM Pressfit vitamys cup than with the RM Pressfit cup. Li et al^11^ also found significantly lower wear rates with VEPE cups in their meta-analysis. The authors highlighted the superiority of VEPE over conventional PE in terms of prevention of osteolysis, implant loosening, and need for revision surgeries. El-Sahoury et al^38^ demonstrated in a recent ten-year RCT a significant difference in wear between VEPE and HXLPE acetabular liner components that favored VEPE. Additionally, there were no significant differences in wear between 32 mm and 36 mm head sizes. Galea et al^39^ compared the wear pattern of VEPE with that of moderately cross-linked PE and reported no significant differences were observed at seven years; however, the authors speculated that the duration may have been inadequate for determining the benefits of VEPE. However, all the mentioned studies used radiostereometric analysis (RSA) for wear measurement. Some other studies that measured wear patterns using EBRA found comparable results. In their multicentre study, Comtesse et al^22^ demonstrated the mid-term wear rates to be below the critical threshold; these rates were independent of the head diameter.

A major goal of contemporary THA is preservation of bone. In this context, two recent studies investigated bone mineral density with the RM Pressfit vitamys cup. In their study, Anderl et al^15^ found the bony changes to remain stable over a five-year period. In their RCT, Brodt et al^16^ observed significantly less bone loss in the polar region of the acetabulum with an RM Pressfit vitamys cup than with a metal-back modular cup.

In modern arthroplasty, one key factor for a successful implant is a long survival rate. Comparable modular acetabular cups made of moderate cross-linked PE or HXLPE demonstrated excellent long-term survival rates of 98% and 100% after ten years.^40,41^ Mahmood et al^19^ reported the longest duration of follow-up for the cup investigated in the present study: they found an implant survival rate of 98.9% after eight years and nine months. In the current study, no cup-related revisions were observed at a mean duration of ten years and eight months; this indicated a survival rate of 100% and confirmed the excellent results. None of the cups demonstrated aseptic loosening. A lucent line was observed for the first time in one patient at five years’ follow-up; this persisted at the last follow-up. However, this patient did not demonstrate a poorer clinical outcome and increased migration. In their cohort, Galea et al^39^ observed the presence of radiolucency in 34% of patients; this was associated with poorer clinical results and increased migration. In their study on porous titanium-coated cups, Lindgren et al^42^ demonstrated an increased risk of radiolucency and persistent gaps after five years. The clinical relevance of these radiological findings is unclear, as none of the cups were revised due to loosening. In this context, Springer et al^43^ found that an incomplete seating of pressfit cups in zone 2 along with radiolucency may achieve fixation safely and effectively. The cup type investigated in the present study frequently displayed a posterior gap due to the rounded design of the reamers and the aspherical cup shape; this mismatch commonly prompted the interpretation of a lucent line. The lack of cup loosening after ten years supports the conclusions drawn by Springer et al,^43^ who suggested that these cups are stable. We therefore speculate that in our case, this line was more likely to be sclerotic and unrelated to stability.

This study has certain limitations, including the non-randomized design and the lack of a control group. The small sample size represents another significant limitation, as it possibly limited the reliability of survival analysis results. It was also inadequate for assessment of revisions and the associated indications – larger studies are therefore required for assessment. Migration and wear measurement using EBRA equally represent a strength and limitation. The strength lies in that it is a widely accepted technical method for retrospective measurement of migration and wear. However, this method also has a high image failure rate and limited accuracy.^26,44^ Additionally, migration and wear are 3D effects, whereas EBRA is a 2D measurement tool. Alternative methods such as RSA may therefore offer more precise results. However, published results from studies using RSA have not revealed any substantial differences. A larger number of patients in this study did not undergo radiological imaging and had non-standardized radiographs, as follow-up examinations were performed during the COVID-19 pandemic. The patients had limited propensity for in-person examinations, and the majority refused to undergo radiography as they believed that radiographs would offer no additional advantages in an uneventful hip. Finally, the inclusion of patients who underwent THA using different stems caused heterogeneity in the group, and this may have been a potential confounder. The major strength of the present study lies in the long duration of follow-up. However, it is worth noting that ten-year results may not provide a final conclusion for any implant in contemporary arthroplasty.

In conclusion, aseptic cup loosening remains the most common reason for revision THA. The RM Pressfit vitamys cup, which has novel properties, was developed to overcome this issue. The present study confirms the considerable success that may be achieved by an isoelastic monoblock cup composed of VEPE. It also highlights the long-term benefits related to prevention of osteolysis, aseptic loosening, and the need for revision surgeries. The survival rate of 100% at a minimum of ten years indicates excellent results and offers promise for future performance. Extended long-term results and data from larger samples are need to validate our findings and confirm the advantages offered by this cup.

**Take home message**

- The RM Pressfit vitamys cup is an innovative cup with promising properties, which has demonstrated excellent results for the first time in a long-term follow-up, and thus may prevent long-term osteolysis, aseptic loosening, and consequently revision surgeries.

## Data Availability

The dataset generated and/or analyzed during the current study are not publicly available due to the high volume of data, but are available from the corresponding author on reasonable request.
